# Bariatric Surgery Leads to a Reduction in Antibodies to Apolipoprotein A-1: a Prospective Cohort Study

**DOI:** 10.1007/s11695-021-05738-7

**Published:** 2021-12-09

**Authors:** Safwaan Adam, Jan H. Ho, Yifen Liu, Tarza Siahmansur, Zohaib Iqbal, Sabrina Pagano, Shazli Azmi, Shaishav S. Dhage, Rachelle Donn, Basil J. Ammori, Akheel A. Syed, Paul N. Durrington, Rayaz A. Malik, Nicolas Vuilleumier, Handrean Soran

**Affiliations:** 1 Faculty of Biology, Medicine and Health, University of Manchester, Manchester, M13 9PL UK; 2 Department of Endocrinology, The Christie NHS Foundation Trust, Manchester, M20 4BX UK; 3 Cardiovascular Trials Unit, Manchester University NHS Foundation Trust, Manchester, M13 9WL UK; 4 Division of Laboratory Medicine, Diagnostic Department, Geneva University Hospital, 1205 Geneva, Switzerland; 5 Department of Internal Medicine Specialities, Medical Faculty, Geneva University, 1205 Geneva, Switzerland; 6 Department of Diabetes, Endocrinology and Obesity Medicine, Salford Royal NHS Foundation Trust, Salford, M6 8HD UK; 7 Department of Medicine, Weill Cornell Medicine-Qatar, 24144 Doha, Qatar

**Keywords:** Anti-apolipoprotein A-1 autoantibodies, High-density lipoprotein, Weight loss, Obesity, Bariatric surgery, Cardiovascular disease

## Abstract

**Purpose:**

Autoantibodies against apolipoprotein A-1 have been associated with cardiovascular disease, poorer CV outcomes and all-cause mortality in obese individuals. The impact of bariatric surgery (BS) on the presence of circulating anti-apoA-1 IgG antibodies is unknown. This study aimed to determine the effect of bariatric surgery on auto-antibodies titres against Apolipoprotein A-1 (anti-apoA-1 IgG), looking for changes associated with lipid parameters, insulin resistance, inflammatory profile and percentage of excess body mass index loss (%EBMIL).

**Materials and Methods:**

We assessed 55 patients (40 women) before, 6 and 12 months post-operatively. Baseline and post-operative clinical history and measurements of body mass index (BMI), serum cholesterol, triglycerides, high- and low-density lipoprotein cholesterol (HDL-C and LDL-C), apoA-1, highly sensitive C-reactive protein (hsCRP), fasting glucose (FG), glycated haemoglobin (HbA1c) and HOMA-IR were taken at each point. Human anti-apoA-1 IgG were measured by ELISA.

**Results:**

The mean age of participants was 50 years. BS significantly improved BMI, %EBMIL triglycerides, HDL-C, apoA-1, hsCRP, HBA1c, FG and HOMA-IR. Baseline anti-apoA-1 IgG seropositivity was 25% and was associated with lower apoA-1 and higher hsCRP levels. One year after BS, anti-apoA-1 IgG seropositivity decreased to 15% (*p* = 0.007) and median anti-apoA-1 IgG values decreased from 0.70 (0.56–0.84) to 0.47 (0.37–0.61) AU (*p* < 0.001). Post-operative anti-apoA-1 IgG levels were significantly associated with a decreased post-surgical %EBMIL at 1 year.

**Conclusion:**

Bariatric surgery results in significant reduction in anti-apoA-1 IgG levels, which may adversely influence weight loss. The exact mechanisms underpinning these results are elusive and require further study before defining any clinical recommendations.

**Graphical abstract:**

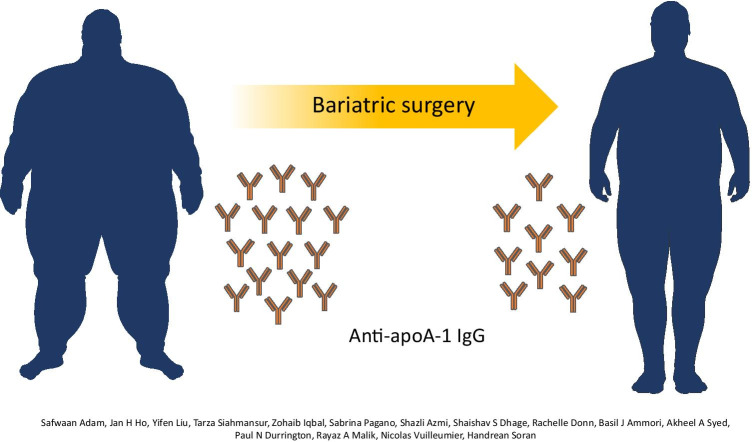

## Introduction

Obesity is an established independent predictor of cardiovascular disease (CVD) [1] and is associated with increased morbidity and mortality [2]. Despite increased recognition of the problem, the prevalence of obesity continues to increase worldwide [3]. Obesity is characterised by a state of chronic low-grade inflammation, a key component in the atherosclerotic process and the development of metabolic complications [4, 5]. Adipocyte hypertrophy [6] marks the beginning of a cascade of complex interlinked pathophysiological processes involving alterations in adipokine production [7], heightened oxidative stress and lipoprotein modification [8], immune system and inflammatory pathway activation [8] and endothelial dysfunction [9], culminating in atherosclerosis and plaque formation.

Over the past decade, autoantibodies against apolipoprotein A-1 (anti-apoA-1 IgG), the principal protein component of high-density lipoprotein (HDL), have emerged as an independent biomarker for cardiovascular disease and mortality [10–12]. Anti-apoA-1 IgG exhibits pro-inflammatory properties through interacting with immune receptors [13] and potentially also exerting a negative effect on HDL function [14]. It has been shown to mediate the process of atherothrombosis and contribute to plaque instability [15, 16]. It is also associated with elevated levels of oxidised low-density lipoprotein (LDL) which is a key component in all stages of atherosclerosis [17]. Anti-apoA-1 IgG was initially identified in patients with autoimmune disease and was linked to atherogenesis and adverse cardiovascular outcomes [18, 19]. Subsequent studies added further support to the link between anti-apoA-1 IgG and cardiovascular disease. Anti-apoA-1 IgG was established as an independent predictor of cardiovascular outcome following myocardial infarction [10], and elevated autoantibody titres were found to be prevalent among patients with acute coronary syndrome [20]. Within the general population, anti-apoA-1 IgG is independently associated with CVD [12] and is an independent predictor of all-cause mortality [11]. In obese patients, anti-apoA-1 IgG has been associated with increased coronary calcium score [21].

Bariatric surgery induces sustained weight loss and long-term reduction in CVD, morbidity and overall mortality [22, 23]. Some of the metabolic benefits of surgery, however, are achieved through weight-independent mechanisms [24]. Post-surgical reductions in markers of inflammation and oxidative stress and improvements in HDL structure and function have been shown in some studies [25–27]. In this study, we sought to determine the impact of bariatric surgery on anti-apoA-1 IgG levels and antibody positivity status. We also examined whether there was any relationship between bariatric surgery–induced changes in anti-apoA-1 IgG levels and cardiovascular risk markers.

Methods.

### Participants and Clinical Measures

Fifty-five patients with severe obesity who underwent bariatric surgery at a tertiary weight management centre were recruited into the study. Patients with acute coronary syndrome within 6 months, acute or chronic infections, human immunodeficiency virus, autoimmune diseases, history of malignancy and those receiving steroid therapy or history of immunosuppressive therapy (including chemotherapy and radiotherapy) were excluded. Study visits were undertaken at baseline, 6 months and 12 months after surgery. The baseline visit was undertaken 6 weeks before surgery, and follow-up visits were within 2 weeks of the 6-month and 12-month time points. Of the fifty-five patients recruited into the study, sixteen were unable to attend the 6-month follow-up visit but all patients attended at 12 months after surgery. At each visit, a clinical history, medication review and clinical examination which consisted of measuring anthropometric measures were undertaken, including body mass index (BMI). The percentage excess in BMI loss (%EBMIL) was calculated using the difference in proportionate change in the BMI in excess of a normal BMI of 24.9 kg/m^2^.

Approval from the local Research and Ethics Committee was obtained. Written informed consent was obtained from all patients prior to participation in this study, and all study assessments were conducted in accordance with the 1964 Helsinki declaration.

### Sample Collection

Venous blood samples were obtained at each study visit between 0900 and 1100 following an overnight fast. Serum and EDTA plasma were isolated by centrifugation at 4 °C within 2 h of collection and aliquots were stored frozen at – 80 °C until laboratory analyses performed at the end of study.

### Biochemical Analyses

Total cholesterol (TC) and triglycerides were measured using the cholesterol oxidase phenol 4-aminoantipyrine peroxidase and glycerol phosphate oxidase phenol 4-aminoantipyrine peroxidase methods, respectively. High-density lipoprotein cholesterol (HDL-C) was assayed using a second-generation homogenous direct method (Roche Diagnostics, Burgess Hill, UK). Apolipoprotein A-1 (apoA-1) was measured using immunoturbidimetry assays with a Cobas Mira analyser (Horiba ABX Diagnostics, Nottingham, UK). Glucose was measured using glucose oxidase-phenol and 4-aminophenazone and hexokinase method. All these tests were performed on a Randox daytona + analyser (Randox Laboratories, Crumlin, UK). The laboratory participated in the RIQAS (Randox International Quality Assessment Scheme; Randox Laboratories, Dublin, Ireland) scheme which is CRC calibrated. Low-density lipoprotein cholesterol (LDL-C) was estimated using the Friedewald formula.

An in-house, antibody sandwich ELISA technique using anti-human C-reactive protein (CRP) antibodies, calibrators and controls (Abcam, Cambridge, UK) was used to measure high-sensitivity CRP (hsCRP).

Glycated haemoglobin (HbA1c) was measured using standard laboratory methods. Insulin was determined using Mercodia ELISA kits (Diagenics Ltd., Milton Keynes, UK). Insulin resistance was quantified using the homeostatic model assessment (HOMA-IR) [28].

### Determination of Anti-apoA-1 IgG Levels

Anti-apoA-1 IgG autoantibodies were measured as previously described [10, 11, 16]. Briefly, MaxiSorp plates (Nunc, Glostrup, Denmark) were coated with purified human-derived delipidated apoA-1 (20 μg/ml; 50 μl/well) for 1 h at 37 °C. After 3 washes, all wells were blocked for 1 h with 2% bovine serum albumin (BSA) in a phosphate buffer solution (PBS) at 37 °C. Samples were then diluted to 1:50 in PBS/BSA 2% solution and incubated for 60 min. Samples at the same dilution were also added to non-coated wells to assess individual non-specific binding. After 6 washes, 50 μl of signal antibody (alkaline phosphatase-conjugated anti-human IgG; Sigma-Aldrich, St. Louis, MO, USA) diluted to 1:1000 in PBS/BSA 2% solution was added to each well and incubated for 1 h at 37 °C. After a further 6 washes, 50 μl of phosphatase substrate *p*-nitrophenyl phosphate disodium (Sigma-Aldrich, St Louis, MO, USA) dissolved in diethanolamine buffer (pH 9.8) was added. Following an incubation period of 20 min at 37 °C, each sample was tested in duplicates and optical density (OD) was determined at 405 nm (Molecular Devices™ Versa Max, Sunny Vale, CA, USA). The corresponding non-specific binding was subtracted from mean absorbance for each sample. The specificity of detection of this ELISA for lipid-low and unmodified apoA-1 was confirmed previously by conventional saturation tests, and liquid chromatography coupled to mass spectrometry [29]. Close to the cut-off value (0.6 OD), the inter-assay coefficient of variation was 9% (*n* = 5), and the intra-assay CV of 5% (*n* = 5).

The cut-off for anti-apoA-1 IgG positivity was determined as previously described [10, 11, 16]. The upper reference range was derived from the 97.5th percentile of reference population for 140 healthy blood donors, and this corresponded with an OD cut-off of 0.64. An index consisting of the ratio between sample OD and positive control OD expressed as a percentage was further calculated to minimise the impact of inter-assay variation. The index value of 37% corresponded with the 97.5th percentile of the normal distribution. Samples with an absorbance value > 0.64 OD and an index value ≥ 37% were considered as seropositive for elevated anti-apoA-1 IgG levels.

### Study Endpoints

The first endpoint consisted of measuring the impact of bariatric surgery on anti-apoA-1 IgG median levels and seropositivity.

The second study endpoint consisted of an evaluation of whether anti-apoA-1 IgG changes could be associated with specific biological and clinical characteristics changes during follow-up.

The third study endpoint consisted of an evaluation of whether baseline and/or post-operative anti-apoA-1 IgG levels were associated with weight loss or 1 year % EBMIL, a key indicator of bariatric success when constantly over 50% on a long-term follow-up [30]. Adjustment was made for other established predictors for bariatric success including age, type of surgery and baseline BMI [31, 32].

### Statistical Analyses

Statistical analyses were performed using SPSS for Mac (Version 23.0, IBM SPSS Statistics, Armonk, NY, USA), and figures were produced using GraphPad Prism for Mac (Version 7.00, GraphPad Software, La Jolla, CA, USA). Normality of data distribution was assessed using the Shapiro–Wilk test. Results were presented as mean with standard deviation (SD) and median with interquartile range (IQR) as appropriate. Comparison between baseline and post-surgery was undertaken using the paired *t*-test for normally distributed variables, Wilcoxon signed-rank test for non-normally distributed variables and McNemar test for categorical variables. For comparison between anti-apoA-1 IgG positive and anti-apoA-1 IgG negative groups, the independent samples *t*-test and Mann–Whitney *U* test were performed for normally distributed and non-normally distributed data, respectively. We also performed a sub-analysis of patients whose antibody status changed from positive to negative and compared them to those patients whose antibody status remained positive. In this sub-analysis, independent *t*-test and Mann–Whitney test were used depending on whether the variable was parametric or non-parametric. Binary logistic regression was used to determine if baseline and post-operative anti-apoA-1 IgG levels could be associated with an EBMIL over 50% at 1 year, whilst controlling for key confounders. Correlations between variables were assessed using Spearman’s analyses. A *p* value of < 0.05 was considered to be statistically significant.

## Results

### Prevalence of Anti-apoA-1 IgG Positivity in Severe Obesity

**Fig. 1:**
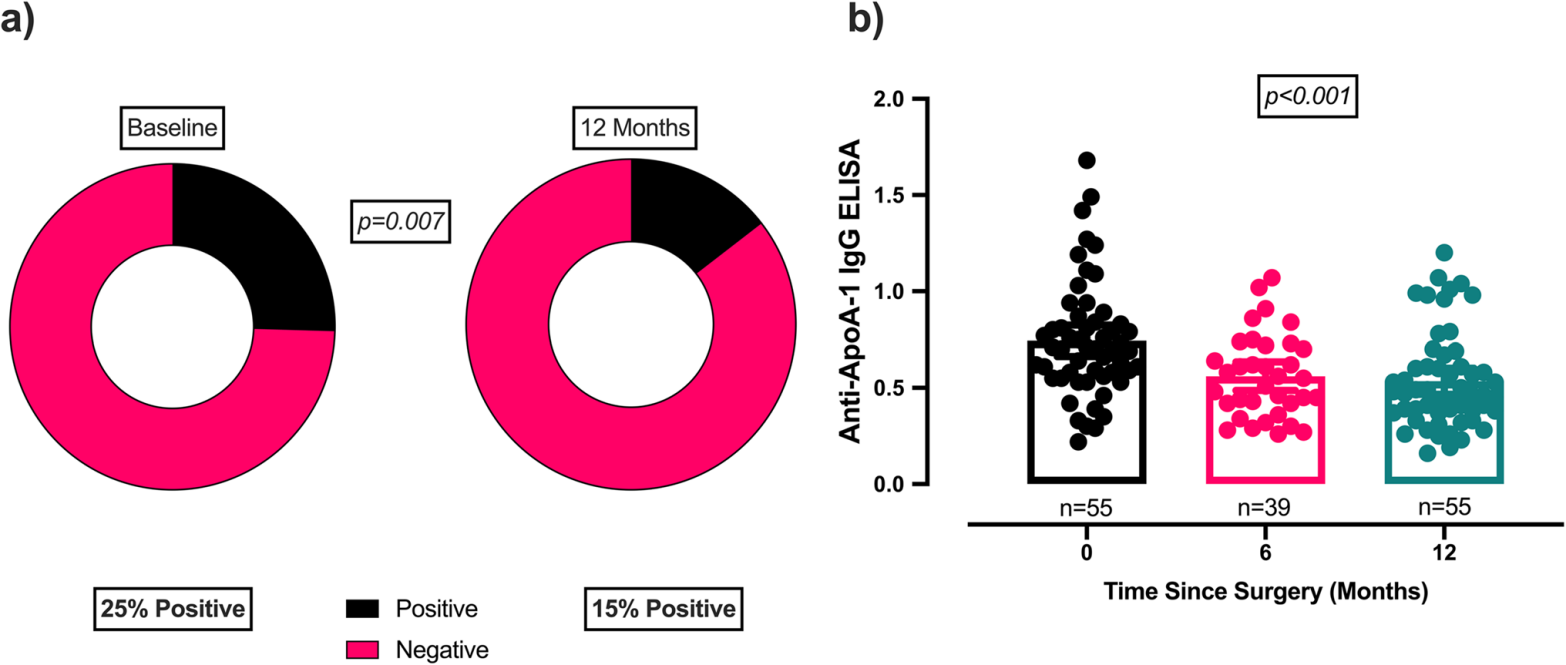
Changes in anti-apoA-1 seropositivity (**a**) and anti-apoA-1 levels before and after bariatric surgery. **a** shows a significant reduction in anti-apoA-1 seropositivity status 12 months after bariatric surgery. **b** shows respective reductions in antibody titres between baseline and 6 months (*p* < *0.001)* and 12 months *(p* < *0.001)* following bariatric surgery. Anti-apoA-1: IgG antibodies to anti-apolipoprotein A-1

Of the 55 patients in the study, at baseline, 14 patients (25%) were positive for autoantibodies against apoA-1 (Table 1 and Fig. 1a). The patients who were autoantibody positive were found to have significantly lower apoA-1 levels (*p* = 0.02) and higher hsCRP levels (*p* = 0.04), but there were no significant differences in the CVD prevalence, type 2 diabetes prevalence, statin use, age, other lipid parameters, HbA1c, fasting glucose, HOMA-IR or BMI (Table 2).

**Table 1:** Pre- and post-operative comparison of variables in patients at baseline, 6 months and 12 months after bariatric surgery

Parameter	Baseline (*n* = 55)	6 months post-surgery (*n* = 39)	12 months post-surgery (*n* = 55)	*p* value
Age (years)	50 (9)			
Female gender (%)	73			
BMI (kg/m^2^)	48.0 (44.0–56.0)	37.0^†††^ (33.0–43.0)	33.0***^§§^ (30.0–38.0)	< 0.001
%EBMIL			64.4 (20.2)	
History of CVD (%)	7			
Type 2 diabetes (%)	53		11	< 0.001
% Statin Use	62		41	< 0.001
Anti-ApoA-1 IgG (AU)	0.70 (0.56–0.84)	0.53^†††^ (0.38–0.72)	0.47*** (0.37 − 0.61)	< 0.001
Anti-ApoA-1 positivity (%)	25	22^†^	15*	0.007
Total cholesterol (mmol/L)	4.05 (3.54–4.66)	4.07 (3.50–4.70)	4.22 (3.62–4.90)	0.332
Triglycerides (mmol/L)	1.46 (1.11–1.87)	1.24 (0.94–1.61)	1.23*** (0.83–1.49)	< 0.001
HDL-C (mmol/L)	1.02 (0.85–1.19)	1.15 (0.94–1.33)	1.21***^§§^ (1.00–1.37)	< 0.001
ApoA-1 (g/L)	1.31 (1.19–1.52)	1.30^†^ (1.14–1.45)	1.30 (1.11–1.50)	0.043
LDL-C (mmol/L)	2.35 (1.91–2.84)	2.21 (1.74–2.72)	2.33 (1.95–3.19)	0.622
hsCRP (mg/L)	6.81 (3.99–13.4)	3.14 (1.03–5.51)	1.34 (0.43–3.22)	< 0.001
HbA1c (%)	6.5 (5.7–7.3)	5.5 (5.2–5.9)	5.4 (5.1–5.7)	< 0.001
HbA1c (mmol/mol)	48 (39–56)	37 (33–41)	35 (32–39)	< 0.001
Fasting glucose (mmol/L)	5.70 (4.88–6.91)	4.93^††^ (4.43–5.93)	4.80*** (4.40–5.53)	< 0.001
HOMA-IR	5.48 (3.41–8.34)	1.95^†††^ (1.15–3.15)	1.69*** (1.17–2.68)	< 0.001

^*^p < 0.05; **p < 0.01; ***p < 0.001 on post hoc testing between baseline and 12 months post-operatively

^†^*p* < 0.05; ^††^*p* < 0.01; ^†††^*p* < 0.001 on post hoc testing between baseline and 6 months post-operatively

^§^*p* < 0.05; ^§§^*p* < 0.01; ^§§§^*p* < 0.01 on post hoc testing between 6 and 12 months post-operatively

Data are represented as mean (standard deviation) or median (interquartile range). Age is representative at the time of surgery; *BMI* body mass index; *CVD* cardiovascular disease; *HDL-C* high-density lipoprotein cholesterol; *ApoA-1* apolipoprotein A-1; *LDL-C* low-density lipoprotein cholesterol; *hsCRP* highly sensitive C-reactive protein; *HbA1c* glycated haemoglobin; *HOMA-IR* homeostatic model of assessment for insulin resistance

**Table 2:** Comparison of variables between patients who tested positive vs negative for anti-ApoA-1 antibodies (at baseline)

	Positive (*n* = 14)	Negative (*n* = 41)	*p*
Female sex (%)	71	73	1.00
Age (years)	51 (11)	49 (9)	0.679
Diabetes prevalence (%)	43	56	0.537
CVD prevalence (%)	14	5	0.266
Anti-Apo-A1 IgG (OD)	1.14 (0.25)	0.61 (0.16)	< 0.001
Total cholesterol (mmol/L)	4.17 (3.60–4.66)	4.03 (3.53–4.95)	0.847
Triglycerides (mmol/L)	1.53 (0.77–1.78)	1.45 (1.18–1.94)	0.374
HDL-C (mmol/L)	0.96 (0.84–1.35)	1.03 (0.82–1.19)	0.931
ApoA1 (g/L)	1.27 (1.09–1.41)	1.33 (1.20–1.55)	0.220
LDL-C (mmol/L)	2.44 (1.99–2.94)	2.26 (1.79–2.92)	0.615
hsCRP (mg/L)	10.4 (5.79–17.5)	5.91 (3.43–9.32)	0.042
HbA1c (%)	6.5 (6.0–6.8)	6.5 (5.7–7.5)	0.862
HbA1c (mmol/mol)	47 (42–51)	48 (39–58)	0.862
Fasting glucose (mmol/L)	5.65 (4.95–6.51)	5.80 (4.82–7.07)	0.517
HOMA-IR (%)	4.30 (3.92–5.74)	5.82 (2.80–9.99)	0.428
BMI (kg/m^2^)	47.5 (43.8–56.3)	48.0 (44.2–55.0)	0.801

Age is representative of age at the time of surgery; *BMI* body mass index; *CVD* cardiovascular disease; *HDL-C* high-density lipoprotein cholesterol; *ApoA-1* apolipoprotein A-1; *LDL-C* low-density lipoprotein cholesterol; *hsCRP* highly sensitive C-reactive protein; *HbA1c* glycated haemoglobin; *HOMA-IR* homeostatic model of assessment for insulin resistance

### The Effect of Bariatric Surgery on Anti-apoA-1 IgG Levels and Autoantibody Positivity Status

All 55 patients underwent bariatric surgery and were followed up 12 months post-operatively. Thirty-nine of these patients attended a 6-month post-operative follow-up visit whilst sixteen patients were unable to do so. Of these 55 patients, 36 underwent Roux-en-Y gastric bypass surgery (RYGB), 11 patients underwent laparoscopic sleeve gastrectomy (LSG) and 8 patients underwent single anastomosis gastric bypass (SAGB). At baseline, 4 out the 55 patients were known to have CVD; 2 of these had an antibody status of positive and 2 were negative for anti-apoA-1 antibodies (Tables 1 and 2). Pre-operatively, 29 patients were known to have Type 2 diabetes which remained in only six patients 12 months post-operatively (Table 1). There was a significant reduction in the median BMI of the entire cohort (*p* < 0.001), with a mean %EBMIL of 64% (Table 1).

At baseline, 14 patients (25%) were positive for anti-apoA-1 antibodies. Of the 14 seropositive patients, 6 patients became seronegative at 12 months, corresponding with a decline in seropositivity prevalence to 15% at after surgery (*p* = 0.007) (Table 1; Fig. 1a). There was also a significant reduction in anti-apoA-1 IgG levels in the entire cohort at both 6 and 12 months post-operatively (*p* < 0.001) (Table 1; Fig. 1b). Additionally, there was a significant increase in HDL-C (*p* < 0.001).Triglycerides (*p* < 0.001), hsCRP (*p* < 0.001), HbA1c (*p* < 0.001), fasting glucose (*p* < 0.001) and HOMA-IR (*p* < 0.001) decreased significantly post-operatively both at 6 and 12 months (Table 1). There was no significant influence from procedure-related effects nor influence of either type 2 diabetes history or statin use in the post-operative results.

### Anti-apoA-1 IgG Associations with Specific Biological and Clinical Characteristics at Baseline and After Surgery

In the entire cohort at baseline (*n* = 55), there was a significant correlation between hsCRP levels and anti-apoA-1 IgG levels (*r* = 0.27; *p* = 0.048), as well as inversely between serum apoA-1 concentration and anti-apoA-1 IgG levels (*r* =  − 0.30; *p* = 0.028) (Table 3).There were no other significant correlations, including between the *percentage change* in measured variables and *percentage changes* in anti-apoA-1 IgG levels.

**Table 3:** Correlation coefficients between anti-apoA-1 IgG and other variables at different time points

	Baseline vs anti-ApoA-1 IgG	6 months post-operative vs anti-ApoA-1 IgG	12 months post-operative vs anti-ApoA-1 IgG
Total cholesterol (mmol/L)	*r* = − 0.07 *p* = 0.59	*r* = − 0.04 *p* = 0.82	*r* = − 0.01 *p* = 0.99
Triglycerides (mmol/L)	*r* = − 0.149 *p* = 0.28	*r* = 0.08 *p* = 0.65	*r* = − 0.08 *p* = 0.60
HDL-C (mmol/L)	*r* = − 0.09 *p* = 0.51	*r* = − 0.12 *p* = 0.48	*r* = 0.002 *p* = 0.99
ApoA-1 (g/L)	*r* = − 0.344 *p* = 0.01	*r* = − 0.27 *p* = 0.11	*r* = − 0.12 *p* = 0.39
LDL-C (mmol/L)	*r* = − 0.04 *p* = 0.80	*r* = 0.001 *p* = 0.99	*r* = 0.008 *p* = 0.95
hsCRP (mg/L)	*r* = 0.272 *p* = 0.04	*r* = 0.09 *p* = 0.60	*r* = 0.19 *p* = 0.18
HbA1c (%)	*r* = 0.137 *p* = 0.32	*r* = 0.09 *p* = 0.62	*r* = 0.07 *p* = 0.63
Fasting glucose (mmol/L)	*r* = − 0.08 *p* = 0.56	*r* = 0.20 *p* = 0.27	*r* = − 0.003 *p* = 0.98
HOMA-IR	*r* = − 0.09 *p* = 0.51	*r* = 0.20 *p* = 0.27	*r* = 0.001 *p* = 0.99
BMI (kg/m^2^)	*r* = 0.107 *p* = 0.44	*r* = 0.22 *p* = 0.20	*r* = 0.12 *p* = 0.38

Spearman’s correlation coefficient between different variables at different time points. Anti-apoA-1 IgG, antibodies to apolipoprotein A-1; HDL-C, high-density lipoprotein cholesterol; ApoA-1, apolipoprotein A-1; LDL-C, low-density lipoprotein cholesterol; hsCRP, high-sensitivity C-reactive protein; HbA1c, glycated haemoglobin; HOMA-IR, homeostatic model of assessment of insulin resistance; BMI, body mass index

Similarly, apart from significant differences in the anti-apoA-1 IgG levels, there were no significant differences in the variables’ percentage change or values at 12 months between seropositive and seronegative patients for anti-apoA-1 IgG.

**Fig. 2:**
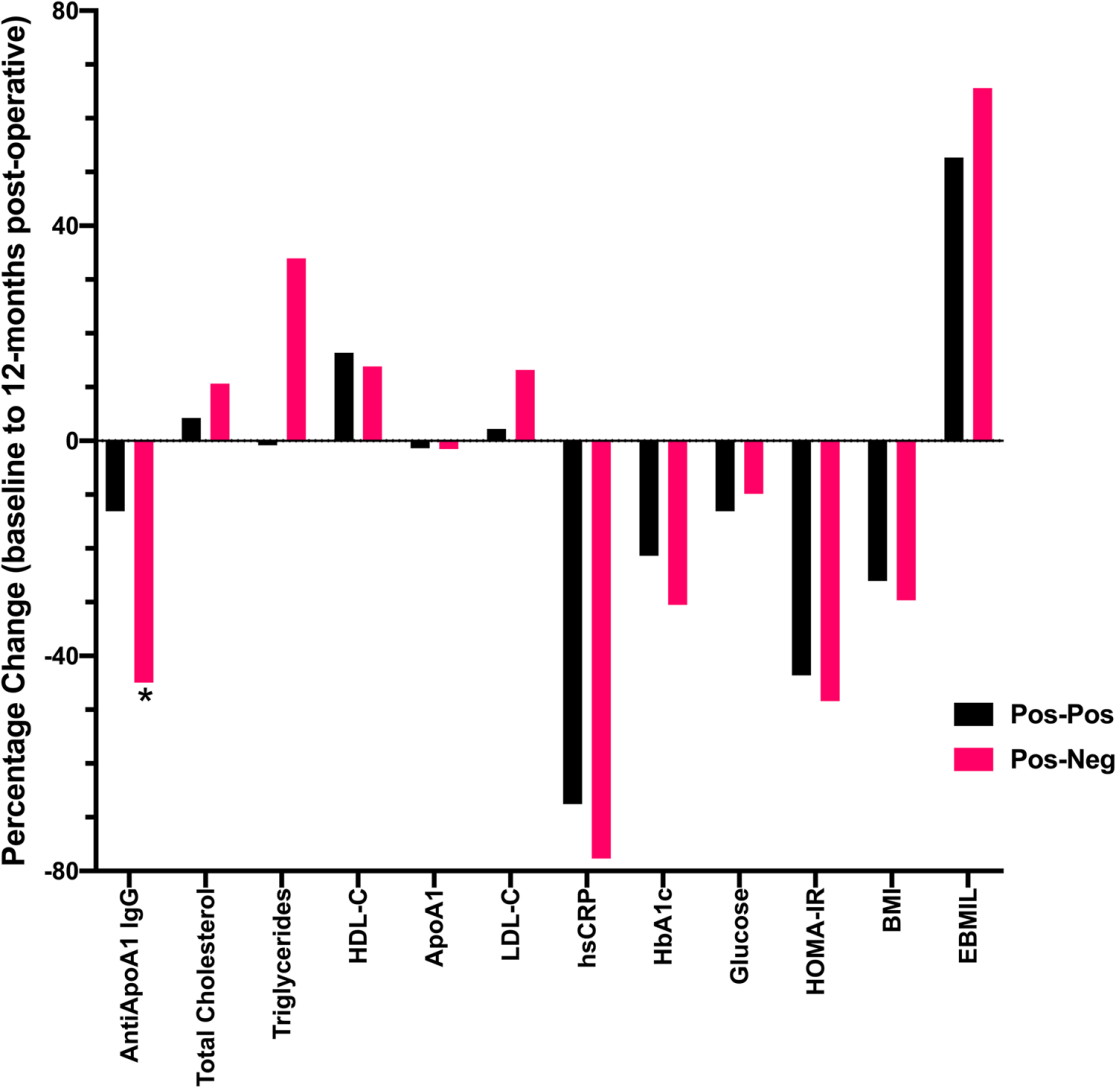
Differences in variables between patients whose antibody status changed from positive to negative compared to those whose antibody status remained positive. There were no statistically significant differences between groups (apart from antibody levels), but there was a trend for greater reductions in hsCRP, HOMA-IR, BMI and increases in %EBMIL, HDL-C and triglycerides. AntiApoA-1, IgG antibodies to apolipoprotein A-1; HDL-C, high-density lipoprotein cholesterol; LDL-C, low-density lipoprotein cholesterol; hsCRP, high-sensitivity C-reactive protein; HOMA-IR, homeostatic model of assessment of insulin resistance; BMI, body mass index; EBMIL, percentage excess BMI loss above 25 kg/m^2^. *p < 0.05

A sub-analysis comparing the 8 patients whose post-operative antibody status remained positive with the 6 patients who became negative for anti-apoA-1 IgG showed, as expected, a significant reduction in anti-apoA-1 IgG levels in the subgroup whose antibody status changed to negative compared to those who remained positive (Fig. 2). Although there was a trend for changes in other measures, none of these reached statistical significance (Fig. 2). There were no significant correlations between anti-apoA-1 IgG levels and other variables seen in either group (positive at baseline who stayed positive and those who changed status to negative) at baseline and 6 and 12 months post-operatively. Similarly, there were no significant correlations between the *percentage chan*ges in variables in either sub-group. The 2 patients with a history of CVD and anti-apoA-1 antibody positivity both changed their status to negative following bariatric surgery.

### Anti-apoA-1 IgG Levels and the Relationship with 1-Year Post-surgery Percentage of Excess Body Mass Index Loss (%EBMIL)

**Fig. 3:**
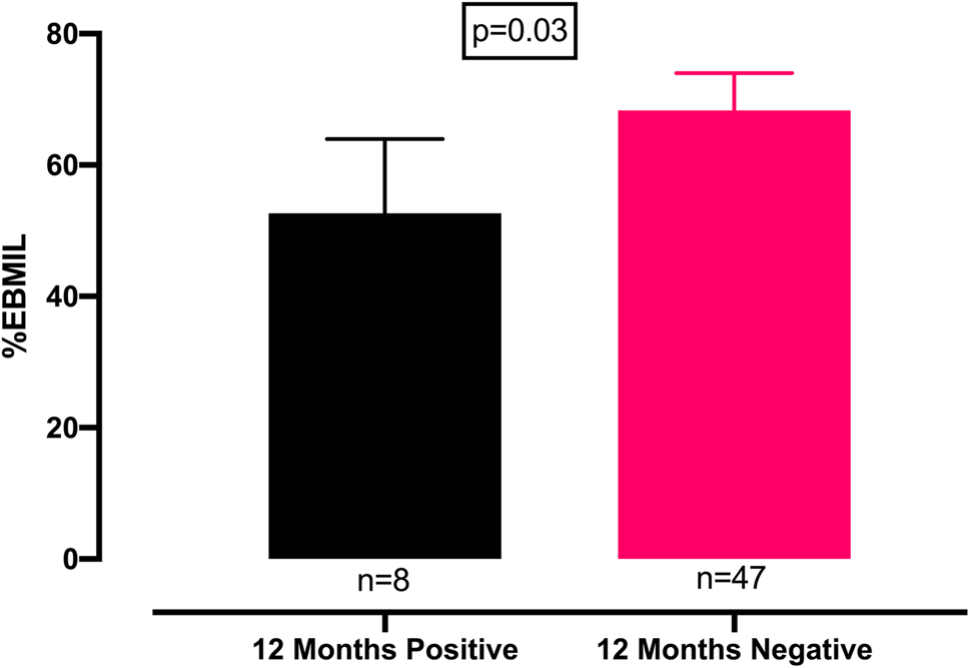
Comparison of %EBMIL in patients whose antibody status was positive or negative 12 months after bariatric surgery. There was a statistically significant difference in the %EBMIL achieved in those with negative antibody titres. %EBMIL: percentage of excess body mass index loss above 25 kg/m^2^

Patients whose antibody status at 12 months post-surgery was negative (*n* = 47) displayed a significantly (*p* = 0.03) greater mean %EBMIL (68%) when compared to those patients (*n* = 8) whose 12-month post-operative antibody status was positive (mean %EBMIL of 53%) (Fig. 3). There were no other significant differences in variables between those who were positive compared to those whose antibody status was negative at 12 months post-bariatric surgery. Logistic regression analysis showed that the 12-month post-operative anti-apoA-1 IgG levels independently exerted an adverse effect on the achievement of a %EBMIL of > 50% (Table 4).

**Table 4:** Binary regression model assessing factors predicting %EBMIL > 50%

Dependent variable %EBMIL > 50% *R*^*2*^ = *0.42; p* = *0.01*		
Covariates	Odds ratio	*p*
Baseline BMI (kg/m^2^)	0.912	0.134
Baseline anti-apoA-1 IgG	3.26	0.404
12-month post-operative anti-apoA-1 IgG	0.003	0.029
Age	0.833	0.007
Procedure	3.17	0.170

A binomial logistic regression was performed to ascertain the effects of age, baseline BMI, 12-month post-operative anti-apo-a1 IgG levels and procedure type on the likelihood that participants had an EBMIL greater than 50%. Of the five predictor variables, only two were statistically significant: age and 12-month post-operative anti-apo-a1 IgG levels

*BMI* body mass index; *anti-apoA-1 IgG* antibodies to antilipoprotein A-1

## Discussion

The novel findings of this study indicate that bariatric surgery reduces anti-apoA-1 IgG levels in severely obese patients as early as 6 months post-operatively. Post-operative anti-apoA-1 IgG levels were significantly associated with a decreased EBMIL at 1 year and were also shown to independently affect the achievement of an %EBMIL of > 50% on regression analysis, suggesting a potential negative impact of sustained presence of these antibodies on weight loss. We did not find any relationships or predictive factors between changes in anti-apoA-1 IgG levels and other biochemical variables.

Interestingly, the prevalence of autoantibody positivity status at baseline in our cohort (25%) was greater than the general population (*n* = 6649) prevalence found in the CoLaus study (19.9%) which comprised a community with a relatively high CVD prevalence [12, 33]. It was also higher than the prevalence in a cohort of patients who were on dialysis for end-stage renal failure, another high-CVD risk group [34]. Furthermore, the baseline anti-apoA-1 autoantibody positivity prevalence in our cohort was almost twofold higher than a cohort of severely obese patients without any history of any metabolic complications [21]. However, after bariatric surgery, the prevalence rates in anti-apoA-1 antibody status were similar in our cohort to the “metabolically healthy” obese patients [21]. This potentially indicates a significant role of obesity as a cardiometabolic risk, mediated via compromising HDL properties. We also show, in keeping with our previous studies, marked improvements in BMI, insulin resistance, hyperglycaemia, inflammation and the lipid profile [25, 35–37].

Obesity has been shown to be an independent risk factor for CVD, and the occurrence of traditional risk factors (chronic hyperglycaemia, hypertension and dyslipidaemia) is higher in obese persons compared to non-obese persons [38]. Additionally, non-traditional risk factors such as inflammation have also been shown to be higher in obese patients such that obesity has been regarded as a chronic inflammatory condition [39]. Importantly, in a previous study done in the setting of another chronic inflammatory condition, rheumatoid arthritis, anti-apoA-1 antibodies were found to be an independent predictor of the presence of CVD [18]. Indeed, in this study, hsCRP levels were higher in the patients whose antibody status was positive compared to negative. Similarly, in our study, there was a moderate correlation between hsCRP levels and anti-apoA-1 IgG levels. Other studies have shown associations between anti-apoA-1 IgG levels, CRP and inflammatory cytokines [12, 13, 16, 40].

Anti-apoA-1 antibodies were previously found to also be independent predictors of all-cause mortality [11] and incident CVD in the general population [39]. Moreover, in otherwise healthy obese persons, anti-apoA-1 antibodies were predictive of coronary artery calcification [21]. Although the numbers were small, in our study, there was a greater proportion of patients with CVD in the patients who tested positive at baseline compared to those who tested negative for anti-apoA-1 antibodies (Table 2). This observation may further underline the role that anti-apoA-1 antibodies have in the pathophysiology of atherosclerotic CVD in obesity and the reduction of these antibodies may be a mechanism by which CVD is reduced post-bariatric surgery.

Although we did not find any relationships between baseline anti-apoA-1 IgG levels and autoantibody positivity status with either weight or BMI, we did observe a greater %EBMIL in patients who were negative compared to those who were positive post-operatively. It is possible that ongoing presence of these antibodies might negatively influence weight loss. Our sample size is relatively small thereby restricting any definitive conclusions, and this observation will need further validation and elucidation in larger clinical studies.

We demonstrated, at baseline, an inverse correlation between anti-apoA-1 IgG levels and apoA-1 levels. This trend has been observed in a previous study examining anti-apoA-1 in type 2 diabetes [42], and although our study was not exclusively in patients with type 2 diabetes, more than half the participants had a history of it. Another recent study in patients with hepatitis C showed that patients who displayed anti-apoA-1 positivity also had significantly lower apoA-1 levels [43]. It has been established that obesity itself alters HDL metabolism (particularly apoA-1) [44], and it may be that the presence of anti-apoA-1 antibodies augments this derangement and therefore enhances CVD risk.

In our analysis, we could not find predictive factors responsible for the reduction in anti-apoA-1 antibody levels. Bariatric surgery improves known systemic CVD risk factors concurrently (e.g. inflammation, dyslipidaemia, hyperglycaemia) [35]. A further longitudinal interventional study in obese persons with both surgical and non-surgical weight loss may help to clarify matters further.

We acknowledge certain limitations in this study. Firstly, the sample size, although allowing us to satisfy the primary aim of the study, did not allow us to perform a detailed analysis of the factors driving changes in anti-apoA-1 antibodies post-bariatric surgery. We also had a greater proportion of women which did not allow us to elucidate sex-specific differences. Furthermore, due to the relatively small numbers, we could not definitively assess for potential procedure related differences. Finally, the prevalence of CVD in our cohort was low and duration of follow-up was relatively short to determine whether the post-bariatric surgery reductions in anti-apoA-1 antibodies have any translatable effect on CVD outcomes.

In summary, we show for the first time that bariatric surgery, through unknown (but likely multifactorial) mechanisms, reduces anti-apoA-1 antibody levels and changes positivity status. Whether this in part explains the reduction in CVD seen in longitudinal cohort studies in patients following bariatric surgery requires further study. The observation that patients whose antibody status was negative had a greater %EBMIL needs to be explored further.

## Data Availability

The datasets generated during and/or analysed during the current study are not publicly available but are available from the corresponding author on reasonable request.
